# Supplementary material for: Chromothripsis during telomere crisis is independent of NHEJ, and consistent with a replicative origin
Source: Genome Res. 2019 May;29(5):737–49. doi: 10.1101/gr.240705.118 (PMC6499312; doi:10.1101/gr.240705.118)
Supplement: Supplemental Material [file supp_gr.240705.118_Supplemental_file_1.zip › contigs/annotated_contigs/DB54/contig.4.DB54_length_772_mean_cov_10.4624352332.docx]

**DB54_length_772_mean_cov_10.4624352332**

```
CTTACATTTCCCTAAGTTTCTGCCGACTAAACTGTGCACACGTTCTCAGGACCTCCTGAAGCTGCGTCACAGGCACTAATCAAAGAACA
 >chr1:238211-238514 - E=2e-170 p=1e-02
CAACCAAGGTGAGTCTCAATCATTTCAAGAAATCTATTTGCAAGGTTAAGGACACACCTGAGAAAAGAACAGAGAACCACAGGAAAAAC

TGTGGTCCGTGCTTTTCCCAAAGATTTTCTGGGGACCTCAGTAAGTAAAGGGGAAAAGTGTGGGTATTGGGGAAAGGGGAAGAAGTGGA

AAAAATGGGTGTGGGTAAATCAGAGGCAAATTGT|TG|AATGAACTGATTTTTCTGTTAAGAACTTTAAATGATAAAAACTCAAATTCT
                                   >chr11:75573162-75573254 + E=2e-43 p=1e-03
ATAGTTTTCTCACTTCCATTTCCTATAAATAAGTTTCTG|CAATTAACTTTAAATTATGCTATCATAAACAATTCATTATATTTAAAAA
                                         >chr10:69212078-69212186 + E=4e-53 p=2e-02
GTTTGTATATTAGTTTATCTTACTACAAAAGAATTCCCAATGGTACCTAACATCTA|ATT|CTTTTGCTCCTTGTAACTTCCTACAGTG
                                                         >chr1:92489-92739 + E=1e-109
TTGTCAGCATCCACATACTTCTCTGTGGGGTTGGTCTCAGAGCCAGGTTACCTTGTCTTAGGTCCAGTGGCACCCTGACTGGCTTGGTG

TCCTTGAACAAGTTACCTAACCTCTCCAAACCTCAGTCCCTCAGTTGTAAAATTAAAAAAAAAAAAAGAAGAAGAAGAGAACCTACGGT

ATAGCAAAGATTTGAAGAAAGAATGAGCTTTTATTATACA|CCATTTTGAAACATTGCCTGACAGG
```
